# Prevalence, trend and contributing factors of geriatric syndromes among older Swedes: results from the Stockholm County Council Public Health Surveys

**DOI:** 10.1186/s12877-018-1018-6

**Published:** 2018-12-29

**Authors:** Yajun Liang, Christian Rausch, Lucie Laflamme, Jette Möller

**Affiliations:** 1 Department of Public Health Sciences, Karolinska Institutet, Widerströmska, 17177 Stockholm, Sweden; 2 University Medical Center Groningen, Department of Health Sciences, Community and Occupational Medicine, University of Groningen, Groningen, The Netherlands

**Keywords:** Foreign-born, Geriatric syndromes, Health inequality, Population study

## Abstract

**Background:**

Evidence is scarce on the trend in prevalence of geriatric syndromes (GS). This study assesses how GS prevalence changes over time in Swedish older community-dwellers by socio-demography, and attempts to highlight factors that may contribute to explain the trend.

**Methods:**

Data from Stockholm County Council Public Health Surveys in 2006, 2010 and 2014 were used. Old adults, aged 65–84 years, with measurements on GS items were identified. Thus, a total of 17,560 participants were selected in 2006 (*n* = 6295), 2010 (*n* = 6733) and 2014 (*n* = 4532). Data on socio-demographics, lifestyles and health status were collected through questionnaires. GS was defined as having at least one of the following: insomnia, urinary incontinence, severe hearing/vision problem, functional decline, fall and depressive disorder. Logistic regression was performed to assess the prevalence trend as well as the change in the associations of sociodemographic factors, health behaviors and chronic disease with GS.

**Results:**

From 2006 to 2014, the prevalence of GS remained stable (*P*_trend_ = 0.54). However, among old adults born outside Nordic countries, it increased significantly from 73.0% in 2006, 78.0% in 2010 to 83.0% in 2014 (*P*_trend_ < 0.001). Furthermore, the association with GS became stronger for born outside Nordic counties (*P*_trend_ < 0.001) and weaker for sedentary lifestyles (*P*_trend_ = 0.004), whereas the association did not change for other sociodemographic factors, health behaviors and chronic disease (all *P*_trend_ > 0.05).

**Conclusions:**

At population level, GS prevalence remained stable at a high level among Swedish old community-dwellers. There are noteworthy differences in GS trend between population groups, in particular to the detriment of older adults born outside Nordic countries.

**Electronic supplementary material:**

The online version of this article (10.1186/s12877-018-1018-6) contains supplementary material, which is available to authorized users.

## Background

As population is aging, health conditions in older adults require increased attention. In that age group, it is not just the single diseases but the concomitant presence of multiple chronic conditions that are increasingly prevalent and lead to high cost of healthcare [1–3]. Geriatric syndromes (GS) are such concomitantly occurring conditions that include pressure ulcers, incontinence, falls, functional decline and delirium [4]. Increased number of GS is associated with greater risk of incident physical disability and lower life satisfaction at the individual level [5, 6].

There are various factors associated with GS including older age (generally > 65 years), unhealthy lifestyles (e.g., alcohol use disorder), functional impairment (e.g., impaired mobility or cognition), prior history of falls, diseases (e.g., multiple comorbidity) and use of medications (e.g., psychoactive medication use) [7–11]. Furthermore, socioeconomic positions (e.g., country of birth, education, occupation, income and wealth) over life course are associated with psychological, physical and cognitive functioning as well as mortality in old age [12–16]. Thus, GS items vary among different sociodemographic groups. For instance, urinary incontinence, falls, functional decline and depression occur more frequently in elderly women than in men [17–19]. People living alone or living in a rental accommodation or assisted living have higher risk of fall injury [17]. The changes in the contributors of GS and their impact on GS over time will contribute to the time trend in GS prevalence. Therefore, examining the trend in the associations between these contributors and GS will aid us to explain the time trends.

However, there is very few evidence on the prevalence trend of GS, especially on the possible contributors of the trend. In this study, we seek to fill in the knowledge gap by first assessing the trend of GS prevalence among old community-dwelling adults and in sub-groups depending on socio-demography, and then examining the changes in associations between possible contributors and GS over time.

## Methods

### Study design and population

This study was based the cross-sectional waves of Stockholm County Council Public Health Surveys (SCCPHS), which were undertaken for the purpose of health and risk factor surveillance as well as for policy formulation, planning and evaluation [20]. To date, four waves of SCCPHS have been completed in 2002, 2006, 2010 and 2014, respectively. In this study, we excluded the wave of 2002 due to a lot of missing information on GS. Therefore, three cross-sectional waves in 2006, 2010 and 2014 were included for assessing the prevalence trend.

For each cross-sectional survey, study sample was selected from the population of Stockholm County based on Swedish Total Population Register using an area-stratified randomization strategy. The eligible population of SCCPHS was those adults aged 18–84 years in 2006 and aged ≥18 years in 2010 and 2014. There are 39 municipalities and urban districts in Stockholm County, and approximately 1300 individuals were sampled from each municipality. Therefore, a total of 50,000 individuals were selected at each survey. For the purpose of this study, we limited the analyses to old adults aged 65–84 years old and excluded the survey in 2002 due to incomplete information on GS. The number of participants (response rate, %) were 6713 (74.5%) in 2006, 7153 (74.1%) in 2010, and 4726 (60.1%) in 2014. After excluding participants with missing information on GS items, a total number of 17,560 old adults were included in the analyses with 6295 old adults in 2006, 6733 in 2010, and 4532 in 2014.

The particular research questions for this study were approved by the Stockholm Regional Ethical Review Board (Dnr 2011/344–31/5, 2013/466–32, 4–1540/2016 and 2016/1932–31/5). For each wave, all participants gave their informed consents by filling in the questionnaire and sending it back.

### Data collection and definitions

Data were collected through postal-based questionnaires for individuals aged 65 years and above [20]. To reduce the non-response rate among immigrants, the questionnaire was translated into six languages (i.e., Arabic, Finnish, Turkish, Farsi, English and Spanish). In case there were persons, who originated from countries where these six languages are official languages and who did not respond to the translated postal questionnaire, a telephone interview in their mother tongue was performed [21]. Similar protocols were used for all three surveys. The self-reported questionnaires comprise about 100 questions covering health parameters including information on socio-demographic factors (such as age, sex, civil status, education, country of origin, living condition and financial status), health behaviors (such as smoking, alcohol intake, physical activity, sedentary lifestyle and nutrition), chronic conditions (such as obesity, hypertension, diabetes, cardiovascular diseases, chronic obstructive pulmonary disease [COPD], hearing impairment, vision problem, functional decline, insomnia, and urinary incontinence) [22].

Based on the previous literature [4, 23], GS was defined as having at least one of the following conditions: insomnia (i.e., having light to heavy sleeping problems), urinary incontinence (i.e., having light to heavy urine leakage), severe hearing problems (i.e., having difficulties in a conversation even with an aid), functional decline (i.e., unable to walk or run 100 m or use stairs), fall (i.e., had at least one injurious fall in the last six months), severe vision problems (i.e., cannot read or distinguish text in a newspaper even with glasses) and depressive symptom (i.e., score of general health questionnaire > 8) [24]. To assess the change of overall burden of GS at population level, both the GS (i.e., having any of the seven above conditions) and the specific GS were included as the variables of interest in the analyses.

Country of origin was grouped into Sweden, other Nordic countries and outside Nordic countries. Furthermore, a group of covariates including socio-demographic factors, health risk behaviors and health conditions were included in the analyses. Civil status was grouped into married, unmarried, divorced and widowed. Education was divided into three groups according to the highest attained level: primary school (≤9 years of education), upper secondary school (10–12 years of education) and university education (≥12 years of education). Type of housing included own, rent and other. Financial stress was defined as having financial hardship in general (e.g., managing the running costs for food, rent, bills, etc.) or in health (e.g., buying medications, going to the dentist or hospital).

Sedentary lifestyles was based on question regarding daily activities and defined as sitting the majority of the time. Current smoking was defined as self-reported of daily smoking currently. Alcohol drinking and nutrition were measured with reference to the last year. For instance, alcohol binge drinking was defined as drinking at least one bottle of wine (or corresponding amount) at least once per week. Unfavorable nutrition was defined as eating greens or fruits less than two times per month. Chronic disease was defined as having at least one of the following conditions including obesity (defined as having a body mass index ≥30 kg/m^2^) and the self-reported physician diagnosis of hypertension, diabetes, heart diseases and COPD.

### Statistical analysis

To describe the characteristics of participants across surveys, linear and logistic regression were performed. To assess the trends in prevalence in GS (i.e., having any of the seven GS items) and in specific GS, binary logistic regression was used with survey time as an independent variable after adjusting for socio-demographic factors, health behaviors and chronic disease. To assess the GS trend in different socio-demographic groups, stratified analyses were performed by age, sex, country of origin and education.

To examine the contributing factors of the trend, binary logistic regression models were used in two steps. First, we assessed the cross-sectional associations between potential contributors (socio-demographic factors, health behaviors and chronic disease) and GS within each survey. Second, we examined the trend in the strength of associations between these factors and GS across surveys, in which an interaction term between year of assessment and individual factors was included into the model together with covariates taking into account the differences in these factors across the three surveys.

Furthermore, the number (%) of participants with missing information was 4 (0.02%) for civil status, 2153 (12.3%) for education, 150 (0.8%) for type of accommodation, 498 (2.8%) for financial stress, 560 (3.2%) for sedentary lifestyle, 2337 (13.3%) for alcohol drinking, 2163 (12.3%) for smoking, 1011 (5.8%) for nutrition and 383 (2.2%) for chronic disease. When these factors were considered as covariates in subsequent analyses, a dummy variable for each of these factors was created to represent those with the missing value. Furthermore, sensitivity analysis with multiple imputation on these missing data was performed to test the robustness of the results.

IBM SPSS Statistics 25 for Windows (IBM SPSS Inc., Chicago, Illinois, USA) was used for all analyses.

## Results

Table 1 shows the characteristics of participants in three surveys. The mean age slightly increased (*P* < 0.001) and the proportion of women varied over time (*P* = 0.011). Across the three surveys participants were more likely to be unmarried, highly educated, current smoking, have unfavorable nutrition and chronic disease, whereas less likely to be widowed, born outside Nordic countries, rent accommodation and have financial stress (all *P* < 0.01). Compare to that in 2006, sedentary lifestyles became less prevalent in 2010 and more prevalent in 2014 (*P* < 0.001). There was no trend in alcohol binge drinking (*P* = 0.306) over time (Table 1).

**Table 1:** Characteristics of participants in the surveys in 2006, 2010 and 2014

Characteristics^a^	2006 (*n* = 6295)	2010 (*n* = 6733)	2014 (*n* = 4532)	*P*
Socio-demography				
Age (years)	72.9 (5.7)	72.2 (5.6)	73.2 (4.8)	< 0.001
Women	3452 (54.8)	3532 (52.5)	2479 (54.7)	0.011
Civil status				
Married	3542 (56.3)	3966 (58.9)	2709 (59.8)	< 0.001
Unmarried	419 (6.7)	503 (7.5)	372 (8.2)	
Divorced	1120 (17.8)	1281 (19.0)	816 (18.0)	
Widowed	1214 (19.3)	983 (14.6)	631 (13.9)	
Country of origin				
Sweden	5194 (82.5)	5624 (83.5)	3861 (85.2)	0.004
Other Nordic countries	523 (8.3)	501 (7.4)	312 (6.9)	
Outside Nordic countries	578 (9.2)	608 (9.0)	359 (7.9)	
Education				
University education	1276 (29.8)	2123 (32.0)	1708 (38.0)	< 0.001
Upper secondary school	1758 (41.1)	2714 (40.9)	1809 (40.3)	
Primary school	1247 (29.1)	1796 (27.1)	976 (21.7)	
Type of accommodation				
Own	4302 (68.7)	4966 (74.3)	3512 (78.7)	< 0.001
Rent	1757 (28.0)	1555 (23.3)	873 (19.6)	
Other	205 (3.3)	160 (2.4)	80 (1.8)	
Financial stress	745 (12.0)	609 (9.5)	361 (8.2)	< 0.001
Health behavior				
Sedentary lifestyle	840 (13.7)	765 (11.7)	647 (14.9)	< 0.001
Alcohol binge drinking	491 (9.1)	509 (8.6)	321 (8.2)	0.306
Current smoking	991 (15.8)	911 (13.8)	518 (20.3)	< 0.001
Unfavorable nutrition	419 (6.8)	585 (9.5)	421 (10.1)	< 0.001
Chronic disease	3671 (60.0)	4341 (65.9)	2967 (66.4)	< 0.001

Values are mean (SD) for age and n (%) for others

^a^When the factors with missing values were considered as covariates in subsequent analyses, a dummy variable was created

**Fig. 1:**
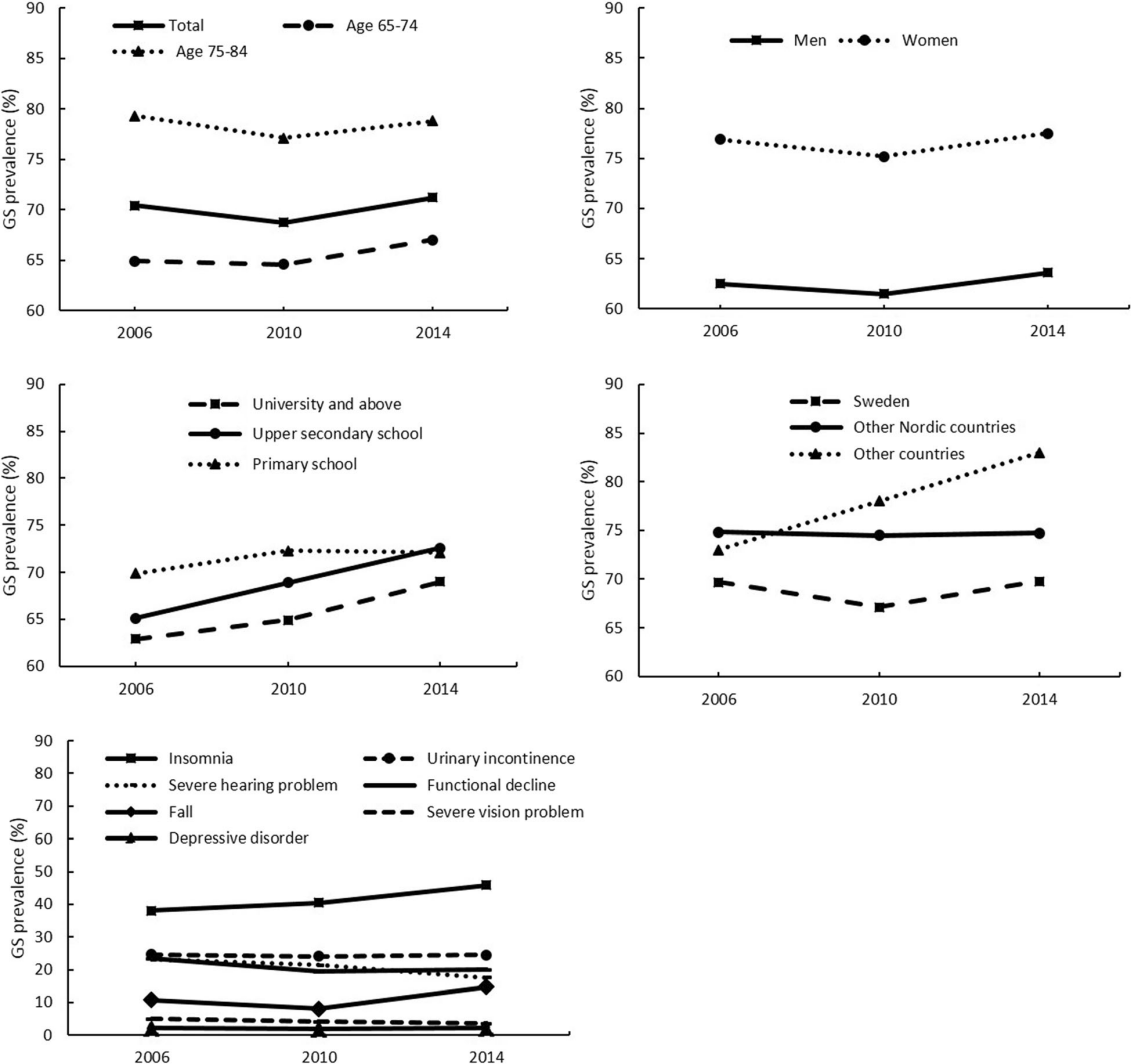
Prevalence of geriatric syndromes (GS) in total participants and subgroups by age, sex, country of origin, education and specific GS

Figure 1, Additional file 1: Table S1 and Table 2 show the trend in prevalence of GS and the trend stratified by age, sex, country of origin, education and specific GS. In all participants, the prevalence of GS did not change significantly over time in the crude model (*P*_trend_ = 0.540). After adjusting for socio-demographic factors, the prevalence slightly increased from 2006 to 2014 (*P*_trend_ = 0.042).

**Table 2:** Trend in prevalence of geriatric syndromes (GS) over time (2006–2014)

		*P*_trend_^a^				
	Annual change (%)	Model 1	Model 2	Model 3	Model 4	Model 5
Total	0.10	0.540	0.899	0.042	0.440	0.614
Age (years)						
65–74	0.26	0.094	0.716	0.127	0.486	0.892
75–84	−0.06	0.551	0.997	0.073	0.950	0.453
Sex						
Men	0.14	0.520	0.849	0.144	0.608	0.702
Women	0.08	0.744	0.991	0.174	0.590	0.781
Country of origin						
Sweden	0.01	0.896	0.693	0.260	0.131	0.385
Other Nordic countries	−0.01	0.965	0.452	0.810	0.576	0.200
Outside Nordic countries	1.25	< 0.001	0.001	< 0.001	< 0.001	0.002
Education						
University and above	0.76	< 0.001	0.473	0.234	0.800	0.532
Upper secondary school	0.94	< 0.001	0.040	0.011	0.384	0.079
Primary school	0.28	0.222	0.407	0.848	0.414	0.257
Specific GS						
Insomnia	0.98	< 0.001	< 0.001	< 0.001	< 0.001	< 0.001
Urinary incontinence	−0.01	0.866	0.780	0.977	0.715	0.430
Severe hearing problem	−0.69	< 0.001	< 0.001	< 0.001	< 0.001	< 0.001
Functional decline	−0.42	< 0.001	< 0.001	0.559	0.001	< 0.001
Fall	0.49	< 0.001	< 0.001	< 0.001	0.001	< 0.001
Severe vision problem	−0.18	< 0.001	< 0.001	0.055	0.001	< 0.001
Depressive disorder	0.00	0.953	0.964	0.628	0.652	0.864

^a^Model 1was a crude model. Model 2 was adjusted for age and sex. Model 3 = Model 2 + other socio-demographic factors (e.g., civil status, country of origin, education, type of accommodation and financial stress). Model 4 = Model 2 + health behaviors (e.g., unfavorable nutrition, sedentary lifestyle, alcohol binge drinking, and current smoking). Model 5 = Model 2 + chronic disease (e.g., cardiovascular diseases, COPD, obesity, hypertension and diabetes)

Notably, the prevalence of GS increased dramatically from 2006 to 2014 in the old adults born outside Nordic coutries (*P*_trend_ < 0.01 for all models), whereas no change was found in Swedish-born and those born in other Nordic countries. After stratification by level of education, the prevalence of GS increased over time among those with highest educational level of university education (*P*_trend_ < 0.001) or upper secondary school (*P*_trend_ < 0.001). Among those with highest educational level of secondary school, GS prevalence increased over time after adjusting for socio-demographic factors (*P*_trend_ = 0.011) but no significant change was observed after further adjusting for health behaviors and chronic disease (Fig. 1 and Table 2).

Among the seven specific GS, insomnia was the most common one, followed by urinary incontinence, severe hearing problem, functional decline, fall, severe vision problem and depressive disorder. After adjusting for covariates, the prevalence increased significantly over time for insomnia and fall (both *P*_trend_ < 0.001), whereas decreased significantly for severe hearing problem, functional decline and severe vision problem (all *P*_trend_ < 0.001). However, the prevalence did not change for urinary incontinence and depressive disorder (Fig. 1 and Table 2).

Furthermore, we assessed the trend in specific GS after stratification by country of origin. The results showed that among those born in Sweden, the prevalence increased over time for insomnia and fall (both *P*_trend_ < 0.001), decreased for severe hearing problem, functional decline and severe vision problem (all *P*_trend_ < 0.001), and remained stable for other items; among those born in other Nordic countries, the prevalence decreased for severe hearing problem (*P*_trend_ = 0.027), and severe vision problem (*P*_trend_ = 0.037) but remained stable for other items; among those born outside Nordic countries, the prevalence increased over time for insomnia (*P*_trend_ < 0.001) but decreased for functional decline (*P*_trend_ = 0.026), no trend was found for other items (Additional file 1: Table S2).

Table 3 shows the trend in cross-sectional associations between various factors and GS. Within each survey, after adjusting for other covariates, the odds ratios of GS were significantly higher in those aged 75–84 years old (vs. those aged 65–74 years), women (vs. men), widowed (vs. married), born outside Nordic countries (vs. Swedish-born), living in other type of accommodation (vs. own accommodation) and in those having financial stress, sedentary lifestyle, alcohol binge drinking, unfavorable nutrition and chronic disease. Compared to that in 2006, after adjusting for confounders, the association with GS became stronger in later years for born outside Nordic countries compared to Swedish-born (*P*_trend_ < 0.001), whereas the association became weaker for sedentary lifestyles (*P*_trend_ = 0.004) (Table 3).

**Table 3:** The associations of socio-demography, health behaviors and chronic disease with geriatric syndromes

Characteristics	Odds ratio (95% confidence interval)^a^			*P*_trend_^a^
	2006	2010	2014	
Socio-demography				
Age (years)				
65–69	Ref	Ref	Ref	
70–84	1.49 (1.23–1.80)	1.62 (1.43–1.84)	1.68 (1.44–1.95)	0.566
Sex				
Men	Ref	Ref	Ref	
Women	1.90 (1.69–2.15)	1.99 (1.78–2.23)	2.22 (1.93–2.57)	0.201
Civil status				
Married	Ref	Ref	Ref	
Unmarried	1.12 (0.88–1.43)	1.06 (0.86–1.31)	1.02 (0.79–1.32)	0.665
Divorced	1.10 (0.94–1.30)	1.09 (0.94–1.26)	0.87 (0.72–1.06)	0.187
Widowed	1.21 (1.02–1.44)	1.19 (0.99–1.42)	1.27 (1.01–1.59)	0.590
Country of origin				
Sweden	Ref	Ref	Ref	
Other Nordic countries	1.13 (0.91–1.41)	1.17 (0.94–1.45)	1.00 (0.76–1.32)	0.491
Outside Nordic countries	0.92 (0.75–1.14)	1.50 (1.21–1.86)	1.85 (1.37–2.50)	< 0.001
Education				
University education	Ref	Ref	Ref	
Upper secondary school	0.97 (0.83–1.14)	1.09 (0.96–1.24)	1.10 (0.95–1.29)	0.252
Primary school	1.06 (0.89–1.27)	1.12 (0.97–1.30)	0.93 (0.77–1.12)	0.304
Type of housing				
Own	Ref	Ref	Ref	
Rent	1.08 (0.93–1.24)	1.13 (0.98–1.30)	1.18 (0.97–1.43)	0.537
Other	1.30 (0.88–1.93)	1.21 (0.79–1.85)	3.73 (1.57–8.87)	0.151
Financial stress				
No	Ref	Ref	Ref	
Yes	2.14 (1.71–2.68)	2.24 (1.76–2.86)	2.91 (2.04–4.14)	0.130
Health behavior				
Sedentary lifestyle				
No	Ref	Ref	Ref	
Yes	4.03 (3.10–5.23)	2.34 (1.91–2.87)	2.34 (1.86–2.95)	0.004
Alcohol binge drinking				
No	Ref	Ref	Ref	
Yes	1.33 (1.07–1.67)	1.60 (1.28–1.99)	1.45 (1.09–1.93)	0.895
Current smoking				
No	Ref	Ref	Ref	
Yes	1.13 (0.96–1.34)	0.97 (0.82–1.14)	0.94 (0.75–1.18)	0.099
Unfavorable nutrition				
No	Ref	Ref	Ref	
Yes	0.97 (0.75–1.24)	1.41 (1.14–1.74)	1.00 (0.79–1.28)	0.679
Chronic disease				
No	Ref	Ref	Ref	
Yes	1.51 (1.34–1.70)	1.59 (1.42–1.78)	1.55 (1.34–1.79)	0.756

^a^Adjusting for other covariates in the table

The sensitivity analysis from data imputation of missing characteristics showed unchanged results except that the annual change of GS prevalence became smaller in the subgroups by education. Furthermore, no trend was found for GS prevalence among those with education of university and above (Additional file 1: Table S3).

## Discussion

In this study we found that the prevalence of GS remained quite stable during 2006 to 2014 among older community-dwellers in Stockholm. However, the prevalence of GS increased in the old adults born outside Nordic countries compared to that of the Swedish-born ones. This association became stronger over time independent of socio-demographic factors, health behaviors and chronic disease. Furthermore, there was a difference in trend among specific GS. The prevalence increased over time for insomnia and fall, decreased for severe hearing problem, functional decline and severe vision problem, whereas remained stable for urinary incontinence and depressive disorder.

We found that the prevalence of GS remained stable at a high level over time. There was very few evidence from previous studies on the trend of GS prevalence. One study in Swedish older-old adults (aged 77 years and older) assessed the trend in complex health problems defined as having severe problems in two or three domains (diseases/symptoms, mobility, and cognitive/communication) [25]. They found that the prevalence decreased significantly from 1992 to 2002 but remained stable from 2002 to 2011 [25]. However, the comparison between our study and theirs was limited by the difference in study participants (e.g., our participants were younger) and variables of interest (i.e., we included different domains for GS definition). Nevertheless, we both found a stable prevalence over a same period. Regarding of the fastest growing population of old adults in Sweden, there will be an increasing number of people with GS even with a stable prevalence. These findings imply that increasing attention is needed from both clinical and public health perspective to reduce the health burden of Swedish aging population. Moreover, studying the change in numbers of GS might also be interesting to look at the development of GS. Regarding that our study is focusing on population level, future research at individual level is needed to look at the change in number of GS.

Notably, elderly people born outside Nordic countries had a higher prevalence of GS than native Swedes at each survey. This is consistent with the previous findings from the H 70 gerontological and geriatric population studies in Gothenburg, which showed that compared with old native Swedes, 70-year-old migrants reported poorer health status (e.g., mental health, vision and urinary problems and general health) [26]. Furthermore, the prevalence of GS increased significantly from 2006 to later years in the old adults born outside Nordic countries which was not the case for the Swedish-born. One possible reason might be that old people born outside Nordic countries are socially and physically vulnerable due to their lower levels of social contacts, poor living conditions (e.g., housing conditions and economic status), less satisfaction with physical health status and lower levels of emotional functioning than the native Swedes [26, 27]. In addition, foreign-born people tend to use less in-hospital care compared to native Swedes, especially men, as regards symptoms, signs, and ill-defined conditions, injury and poisoning [28]. Further studies are needed to explore the underlying mechanism of increasing burden of GS among those elderly people born outside Nordic countries.

The prevalence of specific GS varied greatly from about 2% for depressive disorder to around 40% for insomnia. Although the prevalence of GS remained stable over time, different trends were found for specific GS. These findings raise the necessity of more emphasis on the prevention of those specific GS with high prevalence or increasing burden over time (e.g., insomnia and fall). At the same time, we have to keep in mind that GS are a mixture of multiple conditions that interact with each other with shared risk factors and shared pathophysiologic mechanisms, such as multisystem dysregulation, inflammation, sarcopenia and atherosclerosis [4, 29]. The interactions between specific GS items may result in further functional impairments and higher disease burden [2]. Thus, the prevention on GS calls for a package intervention strategy through taking into account all of these GS items as a whole rather than just focusing on the ones of high and increasing prevalence.

In addition, we found that many risk factors were associated with GS including older age, being windowed, unfavorable accommodation, financial stress, sedentary lifestyle, alcohol binge drinking and chronic disease. This was consistent with previous studies on risk factors of GS [30]. Moreover, our study showed that the association between sedentary lifestyle and GS became weaker, whereas the association between other factors and GS remained stable over time. This finding suggest that sedentary lifestyle is becoming a less important contributor of GS, and further research is needed to explore other unknown determining factors to reduce GS among Stockholm community-dwellers.

One major strength of this study is the population-based study design with large sample sizes. Second, the study protocol and data collection were consistent across all three surveys. However, this study also has limitations that needs to be acknowledged. First, although the participation rate (60.1–74.5%) was relatively high compared with similar surveys [31], the non-respondents could still affect the study population structure and generalizability of our study findings to the whole population. Second, some migrant groups are under-represented in the SCCPHS, however, efforts have been made to overcome this problem, such as translating the questionnaire and offering telephone support [20, 21]. Third, GS were assessed based on self-reported information, which might underestimate the prevalence of some specific GS due to stigmatization or recall bias. Fourth, the association between various factors and GS were cross-sectional, which cannot indicate the causal relationship. Fifth, our study population only included those aged 65–84 years old and living in Stockholm, thus, caution is needed for the generalizability of our findings to other old population. Sixth, we were unable to assess the effect of length of residence due to the lack of data. Although the length of residence in Sweden is an important factor for health of adult immigrants [32], the question of length of stay is less likely to confound the association among older people that we study herein since most of the immigrants in our study originated from Europe, especially Nordic countries, with similar lifestyles before and after immigration.

## Conclusion

The prevalence of GS remained stable at a high level over time in older community-dwellers living in Stockholm, however, with an increasing burden of GS among old adults born outside Nordic countries. Our findings imply that health inequalities due to country of origin persist and become larger during the studied period. Attention is needed to reduce these inequalities in older adults and to achieve healthy equity in ageing in Sweden, which is one of the countries that have the highest proportion of older people in the world together with high level of migration.

## Additional file

Additional file 1: **Table S1.** The prevalence of geriatric syndromes in total participants and subgroups by age, sex, country of origin, education and specific items. **Table S2.** Trend in specific geriatric syndrome (GS) over time (2006–2014) in subgroups by country of origin. **Table S3.** Trend in prevalence of geriatric syndromes (GS) over time (2006–2014) from data imputation of missing characteristics. (DOCX 26 kb)

## Abbreviations

COPD: Chronic obstructive pulmonary disease
GS: Geriatric syndromes
SCCPHS: Stockholm County Council Public Health Surveys
