# Supplementary material for: Prevalence, trend and contributing factors of geriatric syndromes among older Swedes: results from the Stockholm County Council Public Health Surveys
Source: BMC Geriatr. 2018 Dec 29;18:322. doi: 10.1186/s12877-018-1018-6 (PMC6311019; doi:10.1186/s12877-018-1018-6)
Supplement: Supplementary file 1 — Table S1. The prevalence of geriatric syndromes in total participants and subgroups by age, sex, country of origin, education and specific items. Table S2. Trend in specific geriatric syndrome (GS) over time (2006–2014) in subgroups by country of origin. Table S3. Trend in prevalence of geriatric syndromes (GS) over time (2006–2014) from data imputation of missing characteristics. (DOCX 26 kb) [file 12877_2018_1018_MOESM1_ESM.docx]

**Table S1.** The prevalence of geriatric syndromes in total participants and subgroups by age, sex, country of origin, education and specific items

| Characteristics | Prevalence, n (%) | | |
| --- | --- | --- | --- |
|  | 2006 | 2010 | 2014 |
| Total | 70.4 | 68.7 | 71.2 |
| Age (years) |  |  |  |
| Age 65-74 | 64.9 | 64.6 | 67 |
| Age 75-84 | 79.3 | 77.1 | 78.8 |
| Sex |  |  |  |
| Men | 62.5 | 61.5 | 63.6 |
| Women | 76.9 | 75.2 | 77.5 |
| Country of origin |  |  |  |
| Sweden | 69.7 | 67.1 | 69.8 |
| Other Nordic countries | 74.8 | 74.5 | 74.7 |
| Outside Nordic countries | 73 | 78 | 83 |
| Education |  |  |  |
| University and above | 62.9 | 64.9 | 69 |
| Upper secondary school | 65.1 | 68.9 | 72.6 |
| Primary school | 69.9 | 72.3 | 72.1 |
| Specific items |  |  |  |
| Insomnia | 38 | 40.4 | 45.8 |
| Urinary incontinence | 24.6 | 24 | 24.5 |
| Severe hearing problem | 23.1 | 21.4 | 17.6 |
| Functional decline | 23.3 | 19.4 | 19.9 |
| Fall | 10.8 | 8 | 14.7 |
| Severe vision problem | 4.9 | 4.1 | 3.5 |
| Depressive disorder | 2.1 | 1.8 | 2.1 |

**Table S2.** Trend in specific geriatric syndrome (GS) over time (2006-2014) in subgroups by country of origin

|  | Prevalence, n (%) | | |  |
| --- | --- | --- | --- | --- |
| Specific geriatric syndrome | 2006 | 2010 | 2014 | *P*_trend_^a^ |
| Insomnia |  |  |  |  |
| Sweden | 1761 (36.2) | 2117 (38.6) | 1647 (44.0) | <0.001 |
| Other Nordic countries | 217 (45.9) | 228 (46.9) | 160 (53.2) | 0.165 |
| Outside Nordic countries | 255 (47.7) | 305 (52.3) | 197 (58.6) | 0.001 |
| Urinary incontinence |  |  |  |  |
| Sweden | 1234 (25.0) | 1278 (23.8) | 904 (24.5) | 0.579 |
| Other Nordic countries | 116 (23.7) | 124 (26.3) | 69 (23.4) | 0.631 |
| Outside Nordic countries | 116 (12.6) | 131 (24.0) | 82 (24.8) | 0.286 |
| Severe hearing problem |  |  |  |  |
| Sweden | 1193 (23.2) | 1152 (20.6) | 668 (17.4) | <0.001 |
| Other Nordic countries | 124 (24.0) | 123 (24.7) | 51 (16.5) | 0.027 |
| Outside Nordic countries | 125 (21.9) | 160 (26.5) | 73 (20.4) | 0.815 |
| Functional decline |  |  |  |  |
| Sweden | 1132 (22.3) | 1014 (18.5) | 715 (18.8) | <0.001 |
| Other Nordic countries | 124 (24.1) | 110 (22.6) | 76 (25.5) | 0.741 |
| Outside Nordic countries | 178 (31.3) | 144 (24.9) | 93 (26.3) | 0.026 |
| Fall |  |  |  |  |
| Sweden | 523 (10.2) | 417 (7.5) | 553 (14.4) | <0.001 |
| Other Nordic countries | 52 (10.3) | 39 (8.0) | 49 (16.0) | 0.075 |
| Outside Nordic countries | 92 (16.2) | 73 (12.4) | 60 (16.9) | 0.804 |
| Severe vision problem |  |  |  |  |
| Sweden | 232 (4.5) | 207 (3.7) | 126 (3.3) | 0.006 |
| Other Nordic countries | 33 (6.3) | 25 (5.0) | 11 (3.5) | 0.037 |
| Outside Nordic countries | 45 (7.8) | 40 (6.6) | 22 (6.1) | 0.180 |
| Depressive disorder |  |  |  |  |
| Sweden | 99 (1.9) | 84 (1.5) | 75 (2.0) | 0.958 |
| Other Nordic countries | 15 (2.9) | 13 (2.7) | 5 (1.6) | 0.223 |
| Outside Nordic countries | 15 (2.6) | 20 (3.4) | 16 (4.6) | 0.168 |

^a^Adjusted for age and sex.

**Table S3.** Trend in prevalence of geriatric syndromes (GS) over time (2006-2014) from data imputation of missing characteristics

|  | Annual |  |  | *P*_trend_^a^ |  |  |
| --- | --- | --- | --- | --- | --- | --- |
|  | change (%) | Model 1 | Model 2 | Model 3 | Model 4 | Model 5 |
| Total | 0.10 | 0.540 | 0.899 | 0.060 | 0.538 | 0.538 |
| Age (years) |  |  |  |  |  |  |
| 65-74 | 0.26 | 0.094 | 0.716 | 0.066 | 0.581 | 0.968 |
| 75-84 | -0.06 | 0.551 | 0.997 | 0.302 | 0.965 | 0.422 |
| Sex |  |  |  |  |  |  |
| Men | 0.14 | 0.520 | 0.849 | 0.190 | 0.313 | 0.656 |
| Women | 0.08 | 0.744 | 0.991 | 0.204 | 0.819 | 0.712 |
| Country of origin |  |  |  |  |  |  |
| Sweden | 0.01 | 0.896 | 0.693 | 0.421 | 0.200 | 0.324 |
| Other Nordic countries | -0.01 | 0.965 | 0.452 | 0.959 | 0.395 | 0.197 |
| Outside Nordic countries | 1.25 | <0.001 | 0.001 | <0.001 | <0.001 | 0.002 |
| Education |  |  |  |  |  |  |
| University and above | 0.31 | 0.122 | 0.589 | 0.269 | 0.861 | 0.784 |
| Upper secondary school | 0.40 | 0.050 | 0.141 | 0.028 | 0.270 | 0.380 |
| Primary school | 0.31 | 0.127 | 0.207 | 0.615 | 0.207 | 0.078 |
| Specific GS |  |  |  |  |  |  |
| Insomnia | 0.95 | <0.001 | <0.001 | <0.001 | <0.001 | <0.001 |
| Urinary incontinence | -0.01 | 0.803 | 0.754 | 0.854 | 0.710 | 0.424 |
| Severe hearing problem | -0.69 | <0.001 | <0.001 | <0.001 | <0.001 | <0.001 |
| Functional decline | -0.44 | <0.001 | <0.001 | 0.205 | <0.001 | <0.001 |
| Fall | 0.49 | <0.001 | <0.001 | <0.001 | <0.001 | <0.001 |
| Severe vision problem | -0.18 | <0.001 | <0.001 | 0.019 | <0.001 | <0.001 |
| Depressive disorder | 0.00 | 0.911 | 0.911 | 0.408 | 0.963 | 0.740 |

^a^Model 1was a crude model. Model 2 was adjusted for age and sex. Model 3 =Model 2 + other socio-demographic factors (e.g., civil status, country of origin, education, type of accommodation and financial stress). Model 4=Model 2 + health behaviors (e.g., unfavorable nutrition, sedentary lifestyle, alcohol binge drinking, and current smoking). Model 5=Model 2 + chronic disease (e.g., cardiovascular diseases, COPD, obesity, hypertension and diabetes).
